# Supplementary material for: Replication of bacterial plasmids in the nucleus of the red alga Porphyridium purpureum
Source: Nat Commun. 2018 Aug 27;9:3451. doi: 10.1038/s41467-018-05651-1 (PMC6110788; doi:10.1038/s41467-018-05651-1)
Supplement: Supplementary file 1 — Supplementary Information [file 41467_2018_5651_MOESM1_ESM.pdf]

Supplementary Information

Replication of bacterial plasmids in the nucleus of the red alga *Porphyridium purpureum*

Li and Bock

Supplementary Table 1. List of oligonucleotides used in this study.

Primers for vector construction

Vector	Forward primer (5'－3')	Reverse primer (5'－3')	Target sequence
pZL4	CGGCTCGAGATGGCGAAGCTGACGAGCG	CGGGGATCCTCAGTCCTGCTCCTCGGC	*ble*
pZL5	CGGGATCCGTCGACATAGCACGCTAGTATCCTTG	CGGAATTCGTCGACCATCGTTGCGCTACCAGTG	*Tub* promoter
pZL5	CGGGGTACCTAGATCTTGAACCGGTATG	CGGGGTACCTGTAAAGATGCTGTAACCTG	*Tub* terminator
pZL6	CGGGATCCATGGCGAAGCTGACGAGC	CGGGATCCGTCCTGCTCCTCGGCCAC	*ble*
pZL19	CGACTCTAGAGGATCCATGGCGA	GTCCTGCTCCTCGGCCACGA	*ble*
pZL19	GCCGAGGAGCAGGACATGGTGAGCAAGGGCGAGGAG	AGGTACCCGGGGATCCACCCTTGTACAGCTCGTCCATG	*GFP*
pZL22	GCTCTAGATAAACGTCGCGCTAGCTCTCAC	GGGGTACCTTCCTTGTGTTCCACGAAGCAG	*Act* terminator
pZL22	CCAAGCTTGCATGCCTGCAGTTGAGTTCGATCGATTCATGG	GCGACGTTTATCTAGACATGTTGCCTGCACTCCTCGAG	*Act* promoter
pZL22	AGGCAACATGTCTAGAATGTCTGCGTTTGTGAGTG	ATCTCCTTGCCAGACGCCATCGACACGGTC	*ptTP*
pZL22	TACCGAGCTCGAATTCTTGAGTTCGATCGATTCATGG	CCATGATTACGAATTCTTCCTTGTGTTCCACGAAGC	*PAct::TPcp::GFP*
pZL23	CATGCCTGCAGGTCGACTTGAGTTCGATCGATTCATGG	CGTCAGCTTCGCCATGTTGCCTGCACTCCTCGAGCTG	*Act* promoter
pZL23	ATGGCGAAGCTGACGAGC	GTCCTGCTCCTCGGCCAC	*ble*
pZL23	GCCGAGGAGCAGGACTAAACGTCGCGCTAGCTCTCAC	ACGAATTCGAGCTCGGTACCTTCCTTGTGTTCCACGAAGCAG	*Act* terminator
pZL24	TAGTTGCCTGACTCCTGAGGTCTGCCTCGTGAAG	ATGTCGACCTGCAGGCATGCAAGCCACGTTGTGTCTCA	*aphA1*
pZL25	GCAGGAAAGAACATGTAGCTCGCTTGGACTCCTGTTG	CAGACCCCGTAGAAACTAGACCTAGGGTACGGGTT	pSC101 ori
pZL25	TTATCTACACGACGGGGAGTC	TTTCTACGGGGTCTGACGCTC	*bla*

Primers for the generation of hybridization probes

Gene	Forward primer (5'－3')	Reverse primer (5'－3')
ble	CGGGATCCATGGCGAAGCTGACGAGC	CGGGATCCGTCCTGCTCCTCGGCCAC

Tub terminator	TAGATCTTGAACCGGTATG	TGTAAAGATGCTGTAACCTG

Primers for real-time qPCR

Gene	Forward primer **(5'－3')**	Reverse primer **(5'－3')**
Tub coding region	AACGTCTGTCTGTCGAGTATGG	TTCGTTCACAGACATGCTCTC
Tub terminator	TAGATCTTGAACCGGTATG	TGTAAAGATGCTGTAACCTG
EF1α	ACTTCACGACAGCCATTGCCAAG	TGAGAAGATCTTTCCAGAGCAG

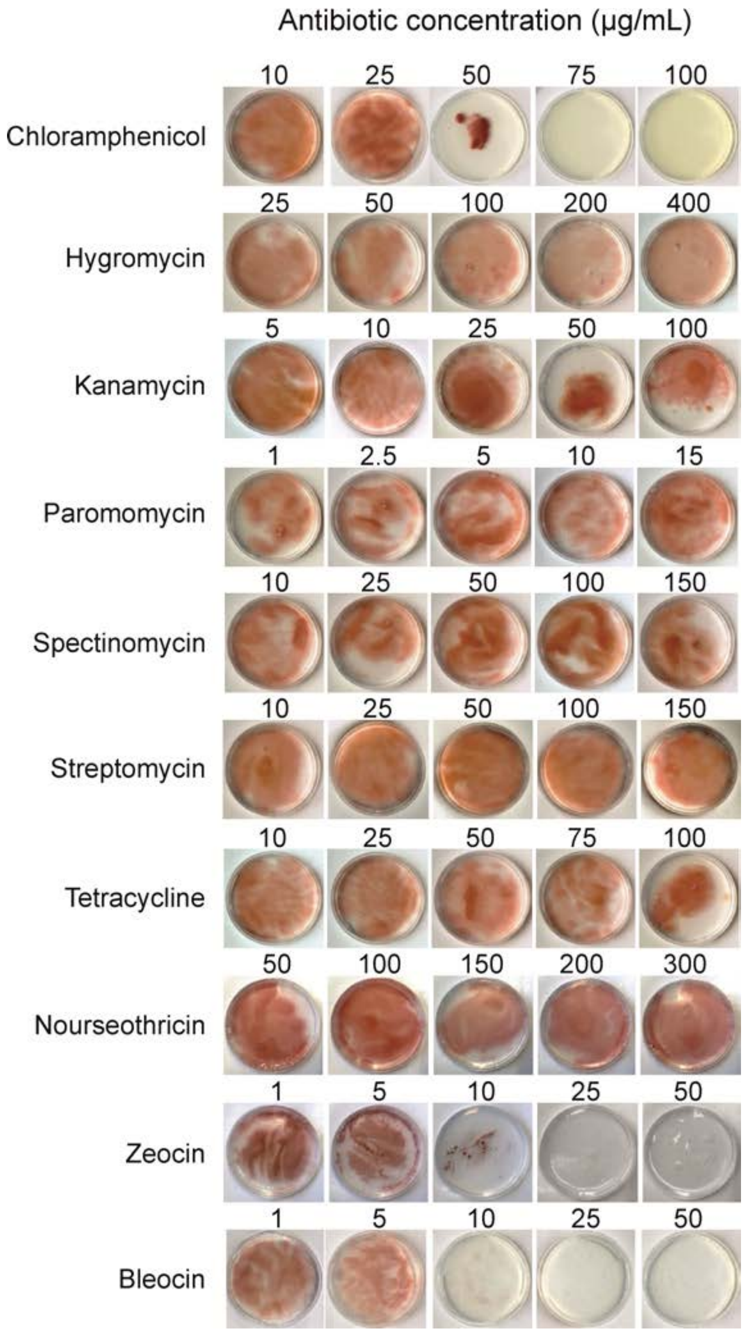

Supplementary Fig. 1 Antibiotic sensitivity tests with the red alga *Porphyridium purpureum* on ASW medium containing different antibiotics. Note that the alga is remarkably tolerant to most antibiotics, but sensitive to the chloroplast translational inhibitor chloramphenicol and the DNA strand break-inducing drugs zeocin and bleocin (both belonging to the bleomycin family of antibiotics).

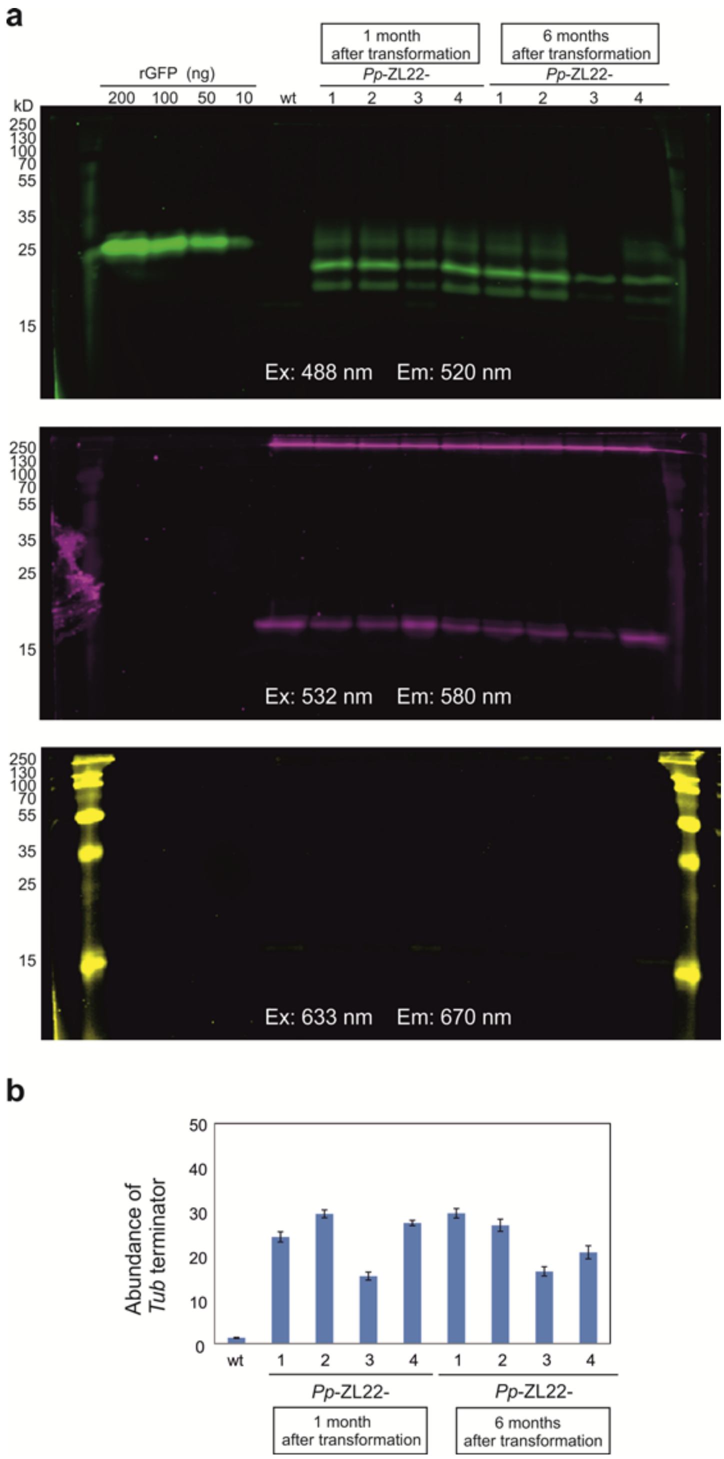

Supplementary Fig. 2 Analysis of algal transformants one and six months after transformation. Strains were maintained under continuous selection. (**a**) In-gel fluorescence analysis of GFP expression. See Fig. 1e. The three individual images are shown here. Upper panel: GFP fluorescence; middle panel: phycoerythrin fluorescence; bottom panel: marker

protein fluorescence; Ex: excitation maximum; Em: emission maximum. (**b**) Measurement of episome copy numbers. Note that copy numbers do not change much and that lower copy numbers (*Pp*-ZL22-3 after 1 and 6 months; *Pp*-ZL22-4 after 6 months) are correlated with lower GFP accumulation levels (cf. panel a).

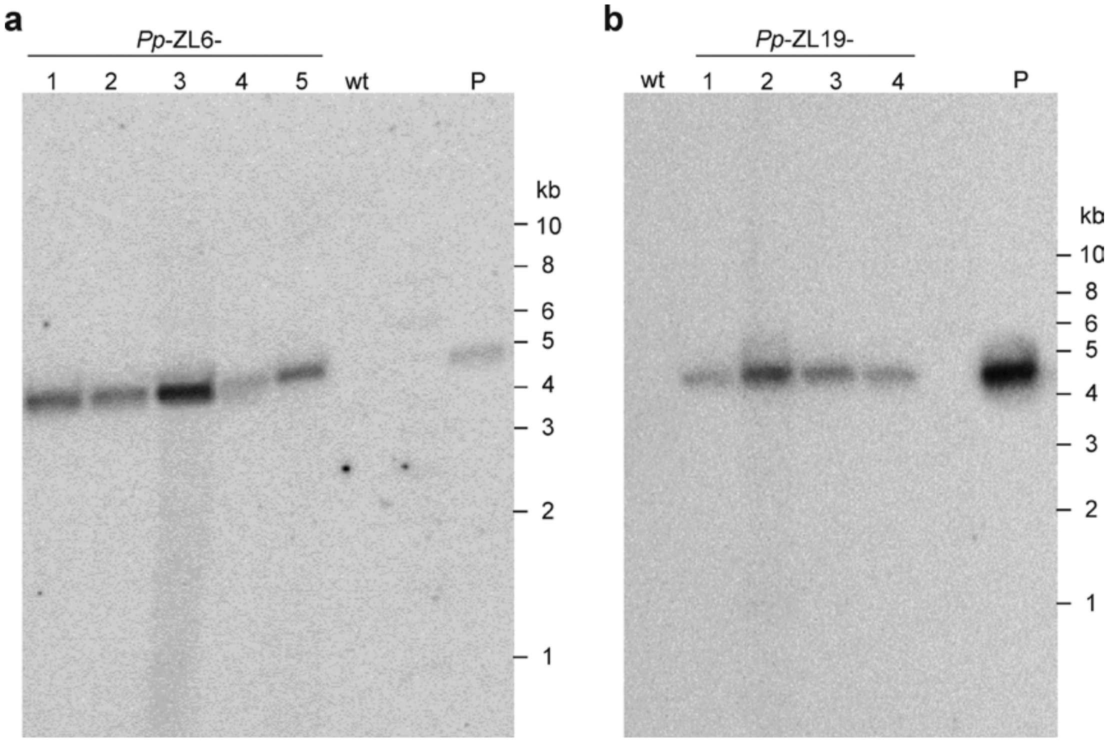

Supplementary Fig. 3 Southern blot analysis of transgenic algal strains generated with transformation vectors pZL6 and pZL19 (cf. Fig. 1b). The entire coding region of the selectable marker gene *ble* was used as hybridization probe. (**a**) Southern blot analysis of *Pp*-ZL6 transformants. Total DNA from five randomly picked transformants and the wild type (wt) was analyzed. Plasmid pZL6 (P) was included as control. All samples were digested with the restriction enzyme EcoRI. (**b**) Southern blot analysis of *Pp*-ZL19 transformants. Samples were digested with the restriction enzyme HindIII.

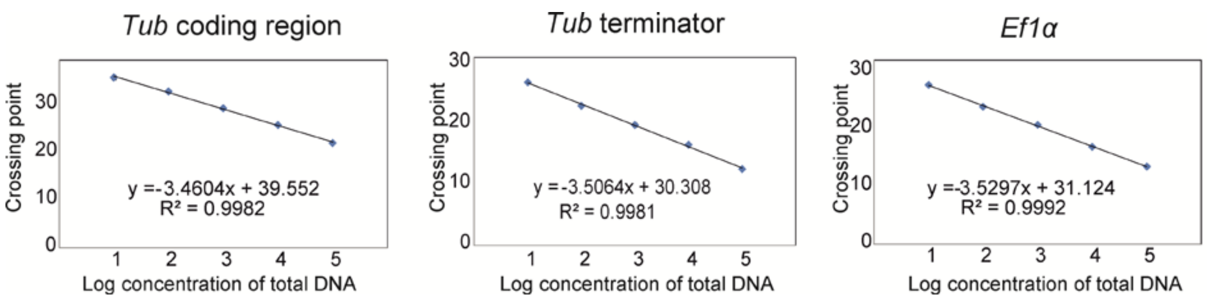

Supplementary Fig. 4. Standard curves for qPCR analysis of the tubulin coding region, the tubulin terminator and the reference gene *EF1α*. The standard curves were generated with a dilution series of total algal DNA. The slope of the standard curve provides a proxy of the efficiency of the real-time PCR. The difference in the slopes of the three standard curves is less than 0.1, indicating that the amplification efficiencies are very similar and the primer pairs can be used for relative quantification of DNA copy numbers.

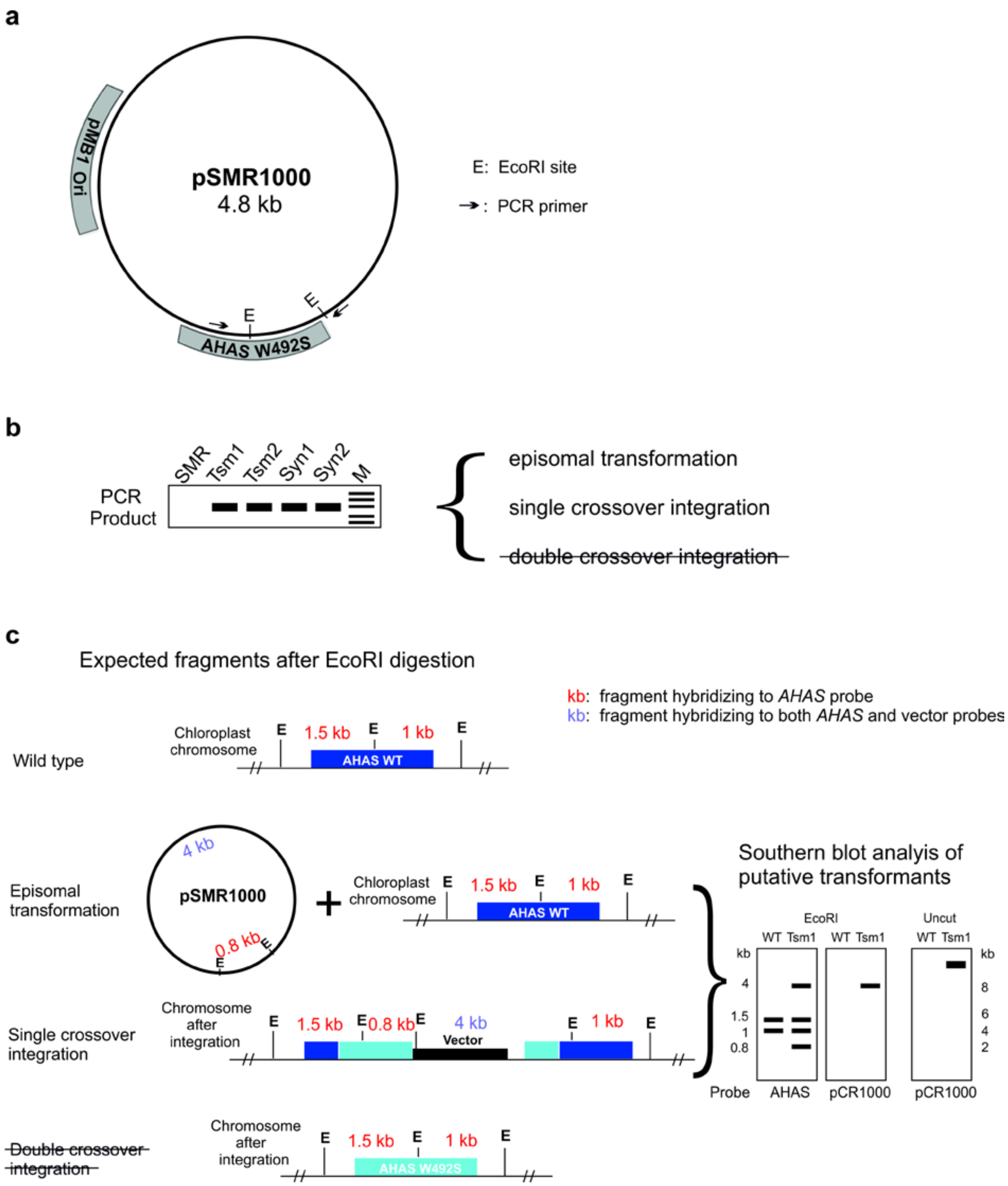

Supplementary Fig. 5. Reassessment of the molecular data from the published report on plastid transformation in *Porphyridium*[1]. (**a**) Schematic map of the plastid transformation vector used. The locations of the PCR primers and the EcoRI restriction sites used for DNA gel blot analysis are indicated. Vector pSMR1000 contains the pMB1 origin of replication. (**b**)

PCR data (gel drawn schematically after Figure 1 in Lapidot et al.[1]). Since both PCR primers bind within the vector sequence, integration by a single crossover event cannot be distinguished from episomal transformation. Since one PCR primer is derived from the vector backbone, amplification of a product in the transgenic strains is incompatible with integration by a double crossover. Note that, due to the high homologous recombination activity in plastids, plastid transformation occurs by double crossovers[2]. Single crossover intermediates are so unstable that they usually are undetectable. (**c**) Reanalysis of the Southern blot data (blot drawn schematically after Figure 2 in Lapidot et al.[1]). Note that, while the data exclude integration by a double crossover, they are equally compatible with episomal nuclear transformation and the unlikely event of integration into the plastid genome by a single crossover.

Supplementary References

1. Lapidot, M., Raveh, D., Sivan, A., Arad, S. & Shapira, M. Stable chloroplast transformation of the unicellular red alga Porphyridium species. *Plant Physiol.* **129**, 7-12 (2002).
2. Bock, R. Engineering plastid genomes: Methods, tools, and applications in basic research and biotechnology. *Annu. Rev. Plant Biol.* **66**, 211-241 (2015).
